# Incidence of Venous Thromboembolism in Newly Diagnosed Glioblastoma and Associated Risk Factors: A Retrospective Chart Review

**DOI:** 10.3390/curroncol32080449

**Published:** 2025-08-10

**Authors:** Duaa Binjabal, Nasser Al Majarafi, Gregory R. Pond, Hal Hirte

**Affiliations:** 1 Department of Oncology, International Medical Centre, Jeddah 23214, Saudi Arabia; djabal@imc.med.sa; 2 Department of Medical Oncology, Juravinski Cancer Centre, Hamilton, ON L8V 5C2, Canada; nasser.al-majarafi@medportal.ca; 3 Department of Oncology, Faculty of Medicine, McMaster University, Hamilton, ON L8S 4L8, Canada; gpond@mcmaster.ca

**Keywords:** glioblastoma, neuro-oncology, venous thromboembolism

## Abstract

This was a single-centre retrospective cohort study of patients diagnosed with glioblastoma (GB) at the Juravinski Cancer Centre (JCC). The charts of 528 patients diagnosed with GB at the JCC from an 8-year period from 1 January 2013, to 31 December 2020, were reviewed. The primary objective was to assess the incidence of venous thromboembolism (VTE) in newly diagnosed GB. The secondary objective was to identify patients at higher risk of developing VTE to understand who might benefit from prophylactic anticoagulation. Data on the following factors were collected: date of diagnosis, time to death or last follow-up, location and size of tumour, degree of resection, presence and location of weakness, performance status, body mass index, comorbidities (hypertension, diabetes, dyslipidemia, smoking history), baseline blood counts, and treatments administered. A total of 111 of the 528 patients (21%) were diagnosed with VTE. Most VTE (87%) occurred within 12 months of diagnosis. A previous cancer diagnosis and recurrence or disease progression were the only factors identified as predictive of a higher risk for developing thrombosis. Newly diagnosed patients with GB have been shown to have a significant risk of developing VTE. Consideration should be given for prophylactic anticoagulation at the time of diagnosis.

## Simple Summary

Patients with glioblastoma are known to be at risk for the development of venous thromboembolism. It is not clear what factors may increase the risk of this, and whether any interventions can be applied to reduce this risk. Our retrospective review of over 500 patients with newly diagnosed glioblastoma over an 8-year period from 2013 to 2020 demonstrated a greater than 20% risk of developing this complication. In most patients, this developed within 12 months of diagnosis, and the only risk factor we identified was a previous diagnosis of cancer. The results of this study will assist clinicians managing these patients, ensuring that there is a high index of suspicion for the development of venous thromboembolism and outlining the risks and benefits of treatments available. A prospective randomized trial to evaluate the efficacy and safety of primary prevention strategies in this high-risk timeframe would be appropriate.

## 1. Introduction

GB is the most common and aggressive primary malignant brain tumour in adults, accounting for approximately 45% of all malignant primary brain tumours. In addition to the direct effects of the tumour, GB patients are at increased risk of developing systemic complications, one of the most significant being VTE. VTE, which includes both DVT and pulmonary embolism (PE), is a known complication in patients with GB and other malignancies. The incidence of VTE in GB patients has been reported to vary widely in the literature, with estimates ranging from as low as 3% to as high as 60% [1,2,3].

Initially, it was believed that the risk of developing VTE was highest during the first few months following diagnosis and surgical intervention [4]. However, more recent studies have demonstrated that the risk remains elevated throughout the course of the disease [1]. This complication significantly impacts the quality of life and overall survival of GB patients, especially when complicated by PE, which is a leading cause of death in cancer patients.

One of the challenges in managing VTE risk in GB patients is whether to use prophylactic anticoagulation. While anticoagulation can reduce the risk of thromboembolic events, it is not routinely recommended for GB patients due to the potential risk of intracranial hemorrhage. Therefore, identifying which patients are at the highest risk of developing VTE could help inform clinical decisions regarding the use of prophylactic anticoagulation and closer monitoring to detect VTE, potentially improving outcomes in this vulnerable population.

Unfortunately, there are presently no reliable tools available to accurately identify high-risk individuals for VTE in GB patients. The only validated risk assessment score for chemotherapy-treated cancer patients with solid tumours, the Khorana score, is not adequately powered for use in patients with brain tumours [5]. Multiple factors are associated with an elevated risk of thrombosis in GB patients, including a combination of patient-related, tumour-related, and treatment-related factors. Patient-related risks include older age, obesity, immobility (particularly due to leg paresis), and a history of VTE [6,7]. Tumour-related factors include larger tumour size and histologic subtype, with GB presenting a higher risk compared to lower-grade gliomas [1,6]. Treatment-related risks include procedures leaving significant residual tumour, such as tumour biopsy and subtotal resection, and the use of corticosteroids or bevacizumab [1,8].

Given the gaps in the current literature, the objective of this study was to examine the incidence of VTE in newly diagnosed GB patients, with a focus on the associated risk factors. By conducting a retrospective chart review, we sought to provide insights into the timing of VTE occurrence and the clinical characteristics that may predispose patients to these events, which might give insight into the potential benefits of prophylactic anticoagulation.

## 2. Materials and Methods

This was a single-centre, retrospective cohort study of newly diagnosed GB patients, 18 years or older, at the Juravinski Cancer Centre (JCC) in Hamilton, Canada. The JCC is a tertiary care centre, with a catchment area of 2.5 million people, located in southeastern Ontario. All patients diagnosed between 1 January 2013 and 31 December 2020 were reviewed. Patients without a pathological diagnosis at the time of data collection were excluded.

### 2.1. Disease and Patients’ Characteristics

Data on the following patient characteristics at diagnosis were collected: age, gender, body mass index (BMI), Karnofsky Performance Status (KPS), functional deficits (weakness), co-morbidities (previous history of VTE or cancer, hypertension, diabetes, dyslipidemia, smoking history), along with the disease characteristics, including tumour anatomic location and size, type of treatment, use of bevacizumab, presence of an intravenous catheter or venacaval filter, date of diagnosis, time to progression, and time to death or last follow-up. Patients whose tumours exhibited an IDH1 or IDH2 mutation were excluded. Laboratory parameters assessed at baseline included leucocyte, hemoglobin, and platelet count. The diagnosis of VTE was confirmed by ultrasound or CT scan along with the type of anticoagulants used and development of bleeding while on treatment. Using the Khorana score [5], patients were grouped by a score of 2 (intermediate risk) versus ≥ 3 (high risk).

### 2.2. Statistical Analyses

Demographic, clinical, surgical, pathological, and radiological data and outcomes were analyzed by descriptive statistics. Time to VTE was calculated using cumulative incidence methods, adjusting for the competing risk of death. Competing risk regression was performed to explore the potential prognostic effect of factors on the cumulative incidence of VTE. Forward stepwise selection was used to construct a multivariable model. Two-sided, 95% confidence intervals were constructed for outcomes of interest. Statistical significance was defined as a *p*-value of 0.05 or less.

### 2.3. Ethics

Ethics approval for this study was obtained from the Hamilton Integrated Research Ethics Board.

## 3. Results

### 3.1. Patient Characteristics

A total of 528 patients diagnosed with GB were included in this study. The patient characteristics are summarized in Table 1. The median age at diagnosis was 65, with 43.6% being female and 56.4% male. A total of 80% of the patients had a KPS of 50 or greater, and 80% of the patients underwent surgical resection, either a gross total resection or subtotal resection, with the remainder having only a biopsy. A total of 80.2% of patients received active treatment, and 9.7% received bevacizumab at some point during their treatments.

### 3.2. Characterization of the VTE Cohort

**Figure 1:**
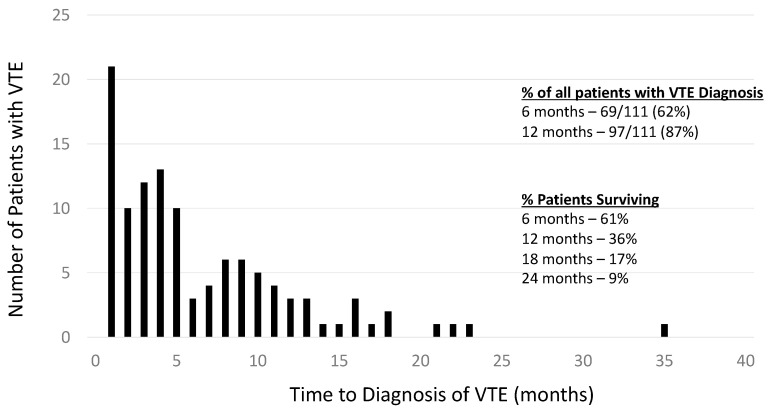
Incidence of VTE over time.

Among the 528 patients studied, 111 patients (21%) were diagnosed with a VTE during the outlined period. Most of the VTE were diagnosed within 12 months of diagnosis of GB (87%), with 62% being diagnosed in the first 6 months (Figure 1).

The cumulative incidence of VTE adjusted for the competing risk of death was 13.5% (95% CI = 10.7% to 16.6%) at 6 months, 18.8% (15.5% to 22.4%) at 12 months, and 23.2% (19.5% to 27.1%) at 24 months.

The location of VTE was as follows: 39 patients (35%) unilateral lower extremity, 8 (7%) unilateral upper extremity, 8 (7%) bilateral lower extremity, 30 (27%) PE, and 25 (23%) DVT and PE. Among these patients, less than 4% of the cases were thought to be catheter related. Intracerebral bleeding developed in 8.3% and other bleeding in 9.5% of the cases while on anticoagulants (see Table 2).

Results of the competing risk regression are shown in Table 3. Having a history of cancer (HR = 1.33, 95% CI = 1.01 to 1.75, *p*-value = 0.045) and recurrence/progression (RP) (HR = 1.61, 95% CI = 1.11 to 2.36, *p*-value = 0.013) were the only statistically significant prognostic factors; however, weakness at baseline (HR = 0.72 for 7 vs. <7, 0.49 to 1.04, *p*-value = 0.080) and platelet count (HR = 0.35 for ≥350 vs. <350, 0.11 to 1.13, *p*-value = 0.079) both approached significance. Using stepwise selection, after RP entered the model, no other factor was statistically significant. A total of 79 of the 528 patients reviewed (14%) had a previous malignancy. Of these, 10 were receiving active therapy for this (3 receiving hormonal therapy and 7 receiving chemotherapy), of whom 4 developed a VTE. Of this group with a previous malignancy, a total of 23 (29%) were diagnosed with VTE.

## 4. Discussion

The development of VTE is a common event in patients with GB. Survival and quality of life are negatively impacted by the development of VTE, particularly if it is complicated by PE. It is associated with a 30% increase in the risk of death within 2 years. The risk of developing VTE is reported to be up to 20% in patients with a brain tumour [1,9].

This retrospective cohort study of 528 GB patients investigated the incidence and clinical characteristics of patients with VTE, providing valuable insights into its occurrence and the associated risk factors in this population. Approximately 21% of newly diagnosed GB patients developed VTE, with 2-year cumulative incidence rates of 23.2%, aligning with rates reported in previous studies [1,2,3,9,10,11,12]. Notably, VTE incidence was highest within the first year of diagnosis, with 62% of cases occurring in the first six months.

In the current study, we examined multiple parameters previously reported as risk factors for VTE in patients with GB. Brandes et al. identified limb paresis and advanced age as significant predictors of VTE in high-grade glioma patients, attributing the increased risk to venous stasis resulting from immobility associated with paresis [6]. Similarly, Marras et al. reported advanced age, extended surgical duration, and larger tumour size as contributors to VTE risk, noting that prolonged surgery may exacerbate blood flow disruption and immobility [1]. Additionally, recent studies have highlighted the role of inflammatory and metabolic markers, such as elevated tissue factors and podoplanin levels in IDH wild-type gliomas, in promoting a hypercoagulable state [13]. Patients with gliomas harbouring IDH1 or IDH2 mutations have been shown to have a decreased risk of thromboembolism, which may be related to a decreased expression of tissue factor, leading to a decrease in coagulation system activation [14,15]. Elevated white blood cell counts and body mass index have also been associated with increased VTE risk, implicating systemic inflammation and metabolic dysregulation as contributing factors [7]. Despite these findings in the literature, our analysis identified only RP and a previous history of cancer as significant prognostic variables for the risk of VTE. The correlation between VTE development and prognosis for survival remains ambiguous [10,16].

Despite the high incidence of thrombosis, routine prophylactic anticoagulation for all patients with GB has not been recommended due to the potential risk of intracranial bleeding or insufficient evidence about its influence on survival. A meta-analysis of ten randomized controlled studies assessed the benefit–risk ratio of several prophylactic VTE measures [17]. This included 1263 patients with primary brain tumours who underwent craniotomy. Prophylactic VTE measures led to a significantly lower risk of VTE without causing a major increase in bleeding. Those receiving unfractionated heparin alone had a larger risk reduction than patients receiving placebo (RR = 0.27, 95% CI 0.1–0.73). Low-molecular-weight heparin (LMWH) together with mechanical prophylaxis demonstrated a lower risk than mechanical prophylaxis alone (RR = 0.61; 95% CI 0.46–0.82).

The PRODIGE trial, a phase III randomized, placebo-controlled trial, evaluated the efficacy and safety of dalteparin for preventing VTE in patients with newly diagnosed malignant glioma [18]. It revealed that primary prophylaxis with LMWH showed a trend toward reduced VTE and increased intracranial bleeding without affecting survival. Unfortunately, the trial was terminated early due to poor accrual and the expiration of the patent on the study medication. The AVERT study examined the preventive benefit of administering a direct oral anticoagulant (DOAC) to cancer patients, including those with GB, at high risk of developing VTE [19]. In this study of 574 cancer patients, apixaban 2.5 mg twice daily was compared to placebo for long-term prophylaxis of VTE. There was a significant reduction of VTE in the apixaban group compared to placebo (4.2% versus 10.2%). However, only a small number of patients in this study had a brain tumour (4.8% in the apixaban group and 3.5% in the placebo group). During the treatment period, major bleeding occurred in six patients (2.1%) in the apixaban group and in three patients (1.1%) in the placebo group (hazard ratio, 1.89; 95% CI, 0.39 to 9.24). However, patients with GB as a subgroup were not analyzed.

As the role of prophylactic anticoagulation for all patients is debatable, identifying those at high risk of developing VTE would potentially allow for the targeted use of preventive anticoagulation to lower the risk of thrombosis. Lim et al. [10] performed a retrospective study aimed at formulating a practical scoring system to predict the risk of VTE in GB patients undergoing chemoradiotherapy. The scoring system included the parameters of age, KPS, smoking history, and hypertension. Patients with a cumulative score of more than 2 points were identified as having a significantly increased risk of symptomatic VTE, with a fivefold higher likelihood compared to those scoring 2 or fewer points. However, since only 115 patients were evaluated in this study, this scoring system requires further validation in larger prospective cohorts to confirm its utility and accuracy in clinical practice.

Newer DOACs, such as rivaroxaban, apixaban, and edoxaban, offer the advantage of oral administration without the need for frequent monitoring. These agents have demonstrated efficacy comparable to LMWH in the treatment and prophylaxis of VTE in cancer patients [18,19]. However, the risk of significant bleeding is slightly higher with DOACs [20,21,22]. This has led to cautious exploration of DOACs in GB, a population at heightened risk for intracranial hemorrhage (ICH). Dubinski et al. conducted a retrospective analysis and found no significant difference in the incidence of major ICH, re-thrombosis, or re-embolism between GB patients treated with DOACs and those treated with LMWH [23]. Additionally, apixaban showed promising safety as a prophylactic agent for VTE in a small cohort of newly diagnosed patients with malignant glioma, with no treatment-related adverse events reported [24].

Despite being one of the largest retrospective studies with 528 patients evaluated and a 21% incidence of VTE, other than a previous diagnosis of cancer, which represented only 14% of our population, we were unable to identify a distinct population at diagnosis of an elevated risk for thrombosis development. Given the limited number of VTE events (111), the findings do need to be interpreted with caution and are a limitation of the study. Consequently, our findings do not support the routine implementation of primary VTE prophylaxis. However, given the high risk of VTE during the first six months after diagnosis, primary prevention could be beneficial during this critical period. A prospective randomized trial would be appropriate to evaluate the efficacy and safety of targeted primary prevention strategies in this high-risk timeframe.

## 5. Conclusions

Newly diagnosed patients with GB have been shown to have a significant risk of developing VTE. Our study is one of the largest retrospective studies assessing the risk of VTE in GB, with 528 patients included. A previous cancer diagnosis and RP were the only factors identified as predictive of a higher risk for developing thrombosis. Given their risk/benefit ratio, consideration should be given to primary prevention of VTE during the first six months after diagnosis with a DOAC. The benefit of preventive treatment would need to be balanced against the risk of bleeding in these patients.

## Figures and Tables

**Table 1:** Patient characteristics at baseline.

Variable		Number Assessed	
Age (years)	Median (range)	528	65 (17, 90)
Gender		528	N (%)
	Female		230 (43.6)
	Male		298 (56.4)
BMI *	Median (interquartile range)	489	27.2 (24.4–31.1)
KPS **		525	N (%)
	30		78 (14.9)
	40		27 (5.1)
	50		34 (6.5)
	60		39 (7.4)
	70		82 (15.6)
	80		118 (22.5)
	90		144 (27.4)
	100		3 (0.6)
Weakness		514	N (%)
	1—unilateral upper		9 (1.8)
	2—unilateral lower		11 (2.1)
	3—bilateral upper		1 (0.2)
	4—bilateral lower		8 (1.6)
	5—hemiplegia/paresis		98 (19.1)
	6—generalized		80 (15.6)
	7—no weakness		307 (59.7)
Location		523	N (%)
	Brainstem		9 (1.7)
	Frontal		158 (30.2)
	Occipital		23 (4.4)
	Parietal		63 (12.1)
	Temporal		141 (27.0)
	Multiple Lobes		129 (24.7)
Number of Lesions		519	N (%)
	Unifocal		410 (79.0)
	Multifocal		109 (21.0)
	Median (interquartile range)	495	4.4 (3.3–5.4)
Surgery		521	N (%)
	Biopsy		103 (19.8)
	Gross total resection		181 (34.7)
	Subtotal resection		237 (45.5)
Treatment		528	N (%)
	Chemoradiation + temozolomide		204 (38.6)
	Chemoradiation		175 (33.1)
	Radiation alone		12 (2.3)
	Temozolomide alone		31 (5.9)
	Missing treatment information		12 (2.3)
	Bevacizumab		51 (9.7)
	No treatment		89 (16.9)

* BMI—body mass index; ** KPS—Karnofsky performance status.

**Table 2:** Incidence of VTE and associated factors.

Variable		Number Assessed	
VTE		528	N (%)
	Yes		111 (21.0)
	No		413 (78.2)
	Missing information		4 (0.8)
Site		111	N (%)
	Arterial thrombosis		1 (0.9)
	Bilateral lower extremity		8 (7.2)
	Unilateral lower extremity		39 (35.1)
	Unilateral upper extremity		8 (7.2)
	Pulmonary embolism		30 (27.0)
	Deep venous thrombosis + pulmonary embolism		25 (22.5)
Intravenous catheter		106	N (%)
	Yes		4 (3.8)
Intra-venacaval filter		106	N (%)
	Yes		8 (7.6)
Intracranial hemorrhage		109	N (%)
	Yes		9 (8.3)
Other bleeding		105	N (%)
	Gastro-intestinal		2 (1.9)
	Hematuria		1 (1.0)
	Intracranial hemorrhage		4 (3.8)
	Epistaxis		2 (1.9)
	Minor bleed		1 (1.0)
	No bleeding		95 (90.5)
Steroids	Yes (%)	510	488 (95.7)
Anticoagulation	Yes (%)	500	73 (14.6)
Antiplatelet agents	Yes (%)	503	56 (11.1)
Survival		528	% (95% CI)
	6-months		73.7 (69.6, 77.4)
	1-year		53.9 (49.3, 58.3)
	2-year		26.3 (22.1, 30.8)
	5-year		8.5 (5.0, 13.0)
Cumulative Incidence of VTE			N (%) Events
	Total	528	111 (21.0)
	6-months		13.5 (10.7, 16.6)
	1-year		18.8 (15.5, 22.4)
	2-year		23.2 (19.5, 27.1)

**Table 3:** Prognostic factors for development of VTE.

Variable	Comparator	*n*	Hazards Ratio (95% CI)	*p*-Value
Age	/year	528	1.00 (0.99, 1.02)	0.98
Sex	Female versus Male	528	0.89 (0.61, 1.30)	0.56
KPS *	/10 units	525	0.96 (0.88, 1.05)	0.41
Weakness	None versus any	514	0.72 (0.49, 1.04)	0.080
Diabetes	Yes versus No	528	0.80 (0.46, 1.38)	0.43
Hypertension	Yes versus No	528	0.96 (0.66, 1.40)	0.82
Dyslipdemia	Yes versus No	528	1.15 (0.78, 1.72)	0.48
History of VTE	Yes versus No	528	0.61 (0.16, 2.36)	0.48
Active Smoker	Yes versus No	528	0.88 (0.47, 1.62)	0.67
History of Cancer	Yes versus No	528	1.33 (1.01, 1.75)	0.045
Number of Lesions	Multifocal versus unifocal	519	0.70 (0.42, 1.17)	0.18
Surgery	Gross Total Resection versus other	528	1.24 (0.86, 1.80)	0.25
RP **	Yes versus No	528	1.61 (1.11, 2.36)	0.013
Bevacizumab	Yes versus No	528	1.22 (0.71, 2.10)	0.47
Khorana Score	≥3 versus 2	470	0.78 (0.53, 1.15)	0.20
Platelets (10^9^/L)	≥350 versus <350	505	0.35 (0.11, 1.13)	0.079
Hb (g/L)	<10 versus ≥10	505	0.89 (0.32, 2.43)	0.81
WBC (10^9^/L)	>11 versus ≤11	504	0.85 (0.58, 1.24)	0.39
BMI ***	≥35 versus <35	489	1.17 (0.65, 2.13)	0.60
Multivariable				
RP **	Yes versus No	528	1.61 (1.11, 2.36)	0.013

* KPS—Karnofsky performance status; ** RP—recurrence/progression; *** BMI—body mass index.

## Data Availability

The raw data supporting the conclusions of this article will be made available by the authors on request.

## Abbreviations

The following abbreviations are used in this manuscript:

GB	Glioblastoma
JCC	Juravinski Cancer Centre
VTE	Venous thromboembolism
BMI	Body mass index
DVT	Deep venous thrombosis
PE	Pulmonary embolism
KPS	Karnofsky performance status
CI	Confidence interval
RP	Recurrence/progression
HR	Hazard ratio
LMWH	Low-molecular-weight heparin
DOAC	Direct oral anti-coagulant
ICH	Intracranial hemorrhage
